# Statistical Tables

**Published:** 1851-12

**Authors:** W. L. Sutton

**Affiliations:** Georgetown, Ky.


Art. II.—*Statistical Tables.* By W. L. Sutton, M. D., Georgetown, Ky.

Since the publication of a digest of the census for Scott county, in the July number of this Journal, I have thought that it would be well to elaborate that portion which pertains to cholera, fever, and typhoid fever something more minutely; I have accordingly prepared the following digest. Table A is a classification of the ages, sex and color of those returned as having died of typhoid fever. Table B is an exhibition of those dying of fever simply:

A

*Typhoid Fever.*

| | Whites. M. | Whites. F. | Blacks. M. | Blacks. F. | Total. |
|---|---|---|---|---|---|
| Under 15.. | 1 | 2 | 1 | 2 | 6 |
| 15 to 35.. | 5 | 2 | 8 | 5 | 20 |
| Over 35.. | 3 | 3 | 0 | 2 | 8 |
| Total,.. | 9 | 7 | 9 | 9 | 34 |

B

*Fever.*

| | Whites. M. | Whites. F. | Blacks. M. | Blacks. F. | Total. |
|---|---|---|---|---|---|
| Under 15.. | 0 | 2 | 3 | 2 | 7 |
| 15 to 35.. | 0 | 2 | 1 | 2 | 5 |
| Over 35.. | 0 | 0 | 1 | 0 | 1 |
| | 0 | 4 | 5 | 4 | 13 |

Of the thirty-four cases of typhoid fever, and thirteen of fever, the duration of illness is shown in the following tables, C and D:

C

*Typhoid Fever.*

| | Whites. M. | Whites. F. | Blacks. M. | Blacks. F. | Total. |
|---|---|---|---|---|---|
| Under 10 days, | 3 | 1 | 3 | 4 | 11 |
| 10 to 25 " | 3 | 3 | 2 | 2 | 10 |
| Over 25 " | 3 | 3 | 4 | 3 | 13 |
| | 9 | 7 | 9 | 9 | 34 |

D

*Fever.*

| | Whites. M. | Whites. F. | Blacks. M. | Blacks. F. | Total. |
|---|---|---|---|---|---|
| Under 10 days, | 0 | 1 | 1 | 0 | 2 |
| 10 to 25 " | 0 | 1 | 4 | 1 | 6 |
| Over 25 " | 0 | 2 | 0 | 3 | 5 |
| | 0 | 4 | 5 | 4 | 13 |

Of the typhoid fever cases, one lasted ninety days, one six months, one eight months; one case of fever lasted sixty days, one a hundred and fifty. Of the typhoid fever cases, one is represented as having proved fatal in three days, and two in six days. Casting out the two cases which lasted six and eight months respectively, the average duration with whites was 23.10 days, among the blacks 23.25 days. Of the cases returned as fever simply, the average duration among whites was 22.75 days: among the blacks casting off one case of one hundred and fifty days, the average was 19.5 days. From all which it is presumable, that they were all cases of typhoid fever.

The following table E shows the age, sex and color of those dying of cholera. Table F shows the duration of illness:

E

*Cholera.*

| | Whites. M. | Whites. F. | Blacks. M. | Blacks. F. | Total. |
|---|---|---|---|---|---|
| Under 15 | 3 | 3 | 3 | 1 | 10 |
| 15 to 35 | 8 | 2 | 2 | 6 | 18 |
| Over 35 | 8 | 1 | 8 | 5 | 22 |
| | 19 | 6 | 13 | 12 | 50 |

F

*Duration.*

| | Whites. M. | Whites. F. | Blacks. M. | Blacks. F. | Total. |
|---|---|---|---|---|---|
| 6 hours and less | 4 | 1 | 1 | 0 | 6 |
| 6 to 24 hours, | 8 | 1 | 5 | 12 | 26 |
| 1 to 3 days, | 3 | 1 | 4 | 0 | 8 |
| Over 3 days, | 4 | 3 | 3 | 0 | 10 |
| | 19 | 6 | 13 | 12 | 50 |

The youngest white dying of cholera was five months and four others were of and under two years; five were over fifty years; two blacks were of and under two years; and six were of fifty years and over.

Two of the attacks are represented as having lasted one hour, one two hours, and one three hours. One case is put down as having lasted twenty-one days; but in truth that was the duration of typhoid fever upon which cholera supervened, and proved fatal in a short time. Another is represented as having lasted seventeen days. I know nothing of the case. Another is stated as of eight days' duration. I am of the impression that a paroxysm of neuralgia, to which the patient was subject commenced at that time, and that cholera lasted between two and three days. Three are noted as lasting five days; and in five whites and fifteen blacks, the time is set down at twenty-four hours.

As not altogether unworthy of notice, I propose to append the following paper:

During the year 1843, owing, as I think, to some inquiries respecting the health of our vicinity, I set about collecting some statistics by my own individual labor; but before I had made much progress, my professional duties put a stop to my inquiries. My observations were made as opportunity offered. Although the number embraced is very limited, I propose to digest the tables: the more especially as some items are embraced in these tables, which are not very frequently attended to. Of the whites registered and then living, four hundred and twenty-two were males, and three hundred and sixty were females.—Of these the ages were as follows:

| Under 5 years, | | 5 to 10 | | 10 to 15 | | 15 to 20 | | 20 to 30 | |
|---|---|---|---|---|---|---|---|---|---|
| M. | F. | M. | F. | M. | F. | M. | F. | M. | F. |
| 57 | 48 | 46 | 43 | 46 | 37 | 60 | 47 | 77 | 76 |

| From 30 to 40 | | 40 to 50 | | 50 to 60 | | 60 to 70 | | Over 70 | |
|---|---|---|---|---|---|---|---|---|---|
| M. | F. | M. | F. | M. | F. | M. | F. | M. | F. |
| 66 | 40 | 41 | 29 | 16 | 22 | 11 | 11 | 2 | 7 |

This register embraces one hundred and sixty-four males and one hundred and sixty-six females, who had been parties to marriage: but these parties were not then all alive. Of these there were married,

| Under 20 years, | | 20 to 30 | | 30 to 40 | | 40 to 50 | | Over 50 | |
|---|---|---|---|---|---|---|---|---|---|
| M. | F. | M. | F. | M. | F. | M. | F. | M. | F. |
| 9 | 90 | 125 | 66 | 20 | 9 | 8 | 1 | 2 | 0 |

Average age, males 26.5—females 20.23 years.

Of one hundred and twenty-one males then living in wedlock, sixteen had married twice; one, three times.—Of the hundred and twenty-one females, five are known to have married twice; none three times. There were twelve widowers, of whom two were widowers the second time. There were thirty-four widows, of whom two were a second time married.

It will be seen that a very large majority of the females married under twenty years. One married at fourteen, and six at fifteen years respectively. Of the males, one married at seventeen, and four at eighteen years.—More than two-thirds of the whole number married between twenty and thirty. One man of fifty-five married a girl of sixteen.

These parties collectively had participated in one hundred and eighty marriages, of which six had taken place within the last twelve months. These hundred and eighty marriages had produced six hundred and seventy-seven children, or 3.76 to a marriage. But of these, ten couples had lived so long childless, that the presumption was fair that they would not have any; seven couples had been recently married and had no children. Casting out the seven couples recently married and it would appear, that about one couple in sixteen is barren. The man of fifty-five whose wife was sixteen at marriage, produced ten children. Thirty-nine couples or two-ninths, had seven or more children.

*Births.*—Within the last twelve months, had been born seventeen males, of which one was still-born; twelve females, all alive.

*Deaths.*—There had been twenty-four deaths; twelve males, twelve females. The ages at death were as follows:

| Under 5 years, | | 5 to 10 | | 10 to 20 | | 20 to 30 | | 30 to 40 | |
|---|---|---|---|---|---|---|---|---|---|
| M. | F. | M. | F. | M. | F. | M. | F. | M. | F. |
| 6 | 2 | 1 | 2 | 1 | 2 | 1 | 4 | 1 | 1 |

| From 40 to 50 | | 40 to 60 | | 60 to 70 | | Over 70 | |
|---|---|---|---|---|---|---|---|
| M. | F. | M. | F. | M. | F. | M. | F. |
| 0 | 1 | 0 | 0 | 1 | 0 | 1* | 0 |

It will be seen that the mortality is very large. In truth it was a very sickly year, affecting mainly young adult persons: two-fifths of the whole mortality being of persons between the ages of ten and forty; one-third precisely, was under five, and only three above forty years.

Of the males, 1 died in 35.16; of the females, 1 in 30; of the whole number, 1 in 32.2.

The proportion at different ages was as follows:

*Of Males Living.*

| | | | | | | |
|---|---|---|---|---|---|---|
| Under 5 years, | 57 | 6 | deaths, | or | 1 in | 9.5 |
| From 5 to 10, | 46 | 1 | " | " | 1 " | 46. |
| " 10 to 20, | 106 | 1 | " | " | 1 " | 106. |
| " 20 to 30, | 77 | 1 | " | " | 1 " | 77. |
| " 30 to 40, | 66 | 1 | " | " | 1 " | 66. |
| " 40 to 50, | 41 | 0 | " | " | 0 " | 00. |
| " 50 to 60, | 16 | 0 | " | " | 0 " | 00. |
| " 60 to 70, | 11 | 1 | " | " | 1 " | 11. |
| over 70, | 2 | 1 | " | " | 1 " | 2. |
| Total,........ | 422 | | | | | |

*Aged 94 years.

*Of Females Living.*

| | | | | | | |
|---|---|---|---|---|---|---|
| Under 5 years, | 48 | 2 | deaths, | or | 1 in | 24. |
| From 5 to 10, | 43 | 2 | " | " | 1 " | 21.5 |
| " 10 to 20, | 84 | 2 | " | " | 1 " | 42. |
| " 20 to 30, | 76 | 4 | " | " | 1 " | 19. |
| " 30 to 40, | 40 | 1 | " | " | 1 " | 40. |
| " 40 to 50, | 29 | 1 | " | " | 1 " | 29. |
| " 50 to 60, | 22 | 0 | " | " | 0 " | 00. |
| " 60 to 70, | 11 | 0 | " | " | 0 " | 00. |
| over 70, | 7 | 0 | " | " | 0 " | 00. |
| Total, ........ | 360 | | | | | |

This number is too small to justify any inference. The only thing about it worth notice, is the very large proportion of deaths of females between twenty and thirty years. Indeed that year was particularly fatal to that class.

Six hundred and seventy slaves were registered; three hundred and eighteen males, three hundred and fifty-two females. Of the slaves there were living, and died, as shown by the following table:

| | Under 10 years, | | 10 to 25 | | 25 to 40 | | 40 to 60 | | Over 60 | | Total. |
|---|---|---|---|---|---|---|---|---|---|---|---|
| | M. | F. | M. | F. | M. | F. | M. | F. | M. | F. | |
| Living, | 109 | 92 | 143 | 142 | 38 | 78 | 20 | 35 | 8 | 5 | 670 |
| Deaths, | 5 | 11 | 3 | 4 | 2 | 2 | 3 | 1 | 1 | 2 | 34 |
| Or 1 in..... | 21.8 | 8.37 | 47.6 | 35.25 | 19. | 39. | 6.66 | 35. | 8. | 25. | 19.41 |

The mortality of the slaves thus appears much greater than among the whites. Of the males 1 in 22.7 died; of the females 1 in 17.6. It must be remarked, that the proportion of deaths of male children under ten years is particularly small. Whether there was an oversight in giving all the children, which is not at all unlikely; or whether the sex was erroneously reported; or whether there is any mistake at all, I cannot tell: but I presume there is a mistake some where.

*Births.*—Among the slaves enumerated, forty-one children were born alive, and one still-born; of the forty-one children, nineteen were males—twenty-two females.

We have here a remarkable example, as far as it goes, that longevity and fecundity are in inverse ratio to each other. Thus in a population of seven hundred and eighty-two whites, of whom 1 in 32.22 die annually, we have twenty-nine births, or one to each twenty-seven of population; and in one of six hundred and seventy slaves, of whom 1 in 19.41 die annually, we have forty-two births, or one to each sixteen of population. Or if we compute the females only; then among females, of whom one in thirty die annually, 1 in 12.4 bears a child annually, whilst among females, of whom 1 in 17.6 die annually, 1 in 8.38 brings a child annually.

Of the children born among the slaves a very large majority, 15 per cent. were females. Whether that was accidental, or whether the proportion at birth is different among whites and blacks, I have no means of knowing; but hope that in two or three years our registration will tell us.

Georgetown, Oct. 15, 1851.

